# Factors affecting acute aortic dissection mortality: A multicentre cohort study

**DOI:** 10.1016/j.sipas.2025.100311

**Published:** 2025-09-21

**Authors:** Joshua G. Kovoor, John M. Glynatsis, Nikolaos C. Glynatsis, Domenico Perrotta, Elyssa Chan, Timothy Daniell, Stephen Bacchi, Brandon Stretton, Daksh Tyagi, Joseph N. Hewitt, Angelyn L.W. Khong, Diana U. Siriwardena, David X.H. Ling, Christopher D. Ovenden, Rohan Arasu, Jonathan Henry W. Jacobsen, Suzanne Edwards, Matthew Marshall-Webb, Pramesh Kovoor, Benjamin A.J. Reddi, Justin C.Y. Chan, Fabio Ramponi, Kurian J. Mylankal, Michael G. Worthington, Aashray K. Gupta

**Affiliations:** a The University of Adelaide, Adelaide, South Australia, Australia; b The Queen Elizabeth Hospital, Adelaide, South Australia, Australia; c Ballarat Base Hospital, Ballarat, Victoria, Australia; d Health and Information, Adelaide, South Australia, Australia; e Flinders Medical Centre, Adelaide, South Australia, Australia; f Lyell McEwin Hospital, Adelaide, South Australia, Australia; g Royal Adelaide Hospital, Adelaide, South Australia, Australia; h Flinders University, Adelaide, South Australia, Australia; i University of Newcastle, Newcastle, New South Wales, Australia; j Wagga Wagga Base Hospital, Wagga, New South Wales, Australia; k Fiona Stanley Hospital, Perth, Western Australia, Australia; l The University of Melbourne, Melbourne, Victoria, Australia; m Box Hill Hospital, Melbourne, Victoria, Australia; n Westmead Hospital, Sydney, New South Wales, Australia; o The University of Sydney, Sydney, New South Wales, Australia; p Prince of Wales Hospital, Sydney, New South Wales, Australia

**Keywords:** Aortic dissection, Mortality, Outcomes, Diagnosis, Time, Delay

## Abstract

**Background:**

Acute aortic dissection (AAD) is an emergency associated with high mortality. Timely diagnosis is challenging, and delays may affect patient outcomes. We aimed to identify clinical and temporal factors associated with mortality after AAD.

**Methodology:**

We performed a retrospective cohort study across four tertiary hospitals of type A and type B AADs diagnosed over a 20-year period. The outcomes of the study were in-hospital mortality, 30-day mortality, and mid-term (6-month) mortality. Univariate linear and bivariate logistic regression analyses were conducted to evaluate the relationship between mortality and demographic and clinical factors.

**Results:**

The study included 149 AAD patients. Of these, 103 (69.1 %) were Stanford type A and 46 (30.9 %) Stanford type B. In-hospital mortality was 29.1 % (*n* = 30) for type A vs 10.9 % (*n* = 5) for type B. For type A patients, every one-year increase in age increased odds of in-hospital mortality by 4 % (*p* = 0.0076), and odds of in-hospital mortality were 10.9 times greater with conservative management than surgical (*p* < 0.0001). Patients with type A dissection had odds of in-hospital mortality 3.0 times greater than type B (*p* = 0.0005). 30-day mortality rate was 29.1 % (*n* = 30) for type A dissection vs 10.9 % (*n* = 5) for type B. 6-month mortality rate was 30.1 % (*n* = 31) for type A dissection vs 10.9 % (*n* = 5) for type B. Predictors of 30-day and 6-month mortality were similar to those of in-hospital mortality.

**Conclusion:**

Even with tertiary care AAD carries a high burden of mortality. Those with type A dissections, increased age, and non-surgical management are at an increased risk of mortality.

## Introduction

Acute aortic dissection is a life threatening emergency with high mortality immediately after symptom onset, making swift diagnosis, transport to hospital, and implementation of a management plan crucial [1]. Initial detection can be reliably facilitated by the Aortic Dissection Detection Risk Score, which incorporates high risk conditions, pain features, and physical examination findings [2,3]. When aortic dissection is suspected, Computed Tomography (CT) is the preferred imaging modality for diagnosis [4,5]. Although novel taxonomies have been developed including type of dissection, location of the tear of the primary entry and malperfusion [6]. classification is most commonly via the Stanford system, which splits aortic dissection anatomically into type A (ascending aorta) and type B (dissection arising distal to type A) [7].

Standardized management of AAD is guided by comprehensive clinical practice guidelines issued by leading professional bodies. In Europe, the European Society of Cardiology (ESC) and European Society for Vascular Surgery (ESVS) provide key recommendations. The 2014 ESC Guidelines on the diagnosis and treatment of aortic diseases [5]. updated in part by the 2024 ESC Guidelines for the Management of Peripheral Arterial and Aortic Diseases, underscore the importance of rapid diagnostic imaging, such as CT, and differentiate management strategies based on Stanford classification and the presence of complications [8]. While Type A dissections typically necessitate urgent surgical intervention, uncomplicated Type B dissections are often managed medically, with endovascular repair considered for complicated cases [8]. The ESVS also contributes with specific guidance on descending thoracic aorta diseases and aortic arch surgery [9,10].

Similarly, in the United States, the 2022 ACC/AHA Guideline for the Diagnosis and Management of Aortic Disease provides detailed recommendations for optimal care [11]. These guidelines advocate for prompt diagnosis, often via CT angiography, and emphasize immediate medical stabilization with intravenous β-blockers for all acute aortic syndromes. Surgical repair remains the cornerstone of treatment for Type A dissections, while Type B dissections are typically managed medically unless complications arise, in which case endovascular or surgical intervention is considered [11]. The Society for Vascular Surgery (SVS) and Society of Thoracic Surgeons (STS) have also established reporting standards for Type B aortic dissections to ensure consistent data collection and analysis [12].

To provide a comprehensive context for the study of AAD, it is essential to consider insights from large international registries. The International Registry of Acute Aortic Dissection (IRAD), established in 1996, stands as the largest ongoing prospective registry of AAD, encompassing extensive data from numerous referral centres across 13 countries [13]. IRAD has been instrumental in elucidating the epidemiology, clinical features, management strategies, and outcomes of AAD, significantly informing clinical guidelines globally. For instance, early IRAD data reported an overall in-hospital mortality of 27.4 %, with surgical mortality for Type A dissections at 26 % and medical management mortality for Type B at 10.7 % [13,14]. Over its 25-year history, IRAD has documented a notable decrease in-hospital mortality for both Type A (from 31 % to 22 %) and Type B (endovascular mortality from 9.9 % to 6.2 %) dissections, a trend largely attributed to advancements in surgical approaches for Type A and increased utilization of thoracic endovascular aortic repair (TEVAR) for Type B [[13], [14], [15]].

Despite these improvements in mortality after acute aortic dissection, it still remains high and evidence-based improvement in clinical care is needed. We performed a retrospective multicentre cohort study to determine whether demographic (age, sex), clinical (Stanford type, management approach, first systolic blood pressure, ADDRS, ASA class), and temporal (time to CT diagnosis, time to surgery) factors are independently associated with in‑hospital, 30‑day (short‑term), and 6‑month (mid‑term) mortality.

## Methods

### Study design, setting and population

The inclusion criteria for this retrospective, observational study was: patients aged over the age of 18 years with acute aortic dissection diagnosed on CT scan, who presented to four metropolitan centres in South Australia between 2001 and 2021 (Royal Adelaide Hospital, The Queen Elizabeth Hospital, Flinders Medical Centre, Lyell McEwin Hospital), and who had data available regarding time of CT diagnosis and time of first hospital contact. Both Stanford type A and type B acute aortic dissections were included. After presentation and initial diagnosis on CT, all patients were transferred to a single quaternary, metropolitan centre for admission and care within a specialized cardiothoracic and vascular surgery unit. Ethical approval for this study was obtained from the Central Adelaide Local Health Network Human Research Ethics Committee (reference number: 15,157).

### Data collection and measures

Data were retrospectively extracted from hard-copy and electronic patient medical records. All data were stored in a secure electronic database within research electronic data capture (REDCap) [16]. In accordance with terminology used in cardiovascular outcome research we classify death within 30 days as short‑term, and death between 30 days and six months as mid‑term mortality [[17], [18], [19]]. Six‑month mortality is therefore our mid‑term endpoint. Outcomes of interest included in-hospital mortality, 30-day mortality and 6-month (mid-term) mortality. The relationship between these outcomes and various predictors was investigated, including: time from first hospital contact to diagnosis on CT, Aortic Dissection Detection Risk Score (ADDRS) [2]. American Society of Anaesthesiology (ASA) Classification of physical status [20]. first systolic blood pressure recorded at hospital, method of hospital arrival and Stanford classification of the aortic dissection [7]. A detailed analysis of the temporal relationship of critical events from symptom onset to arrival at quaternary centre, diagnosis and operative intervention were analysed along with relationship to patient characteristics. Investigation and outcome analysis was conducted separately for the total study cohort, patients with type A acute aortic dissection, and patients with type B acute aortic dissection. To minimise selection bias, no prior sample-size calculation was performed - the cohort size (*n* = 149) reflects all eligible consecutive cases during the 20‑year period.

### Statistical analysis

All statistical software was performed using SAS 9.4 (SAS Institute Inc., Cary, NC, USA). Univariate linear and binary logistic regression analyses were conducted to assess association between outcomes and predictors. Age and ADDRS were modelled continuously (per 1‑unit increase), first systolic BP per 10 mmHg, and time intervals per hour. Management approach and Stanford type were categorical. Odds ratios and mean differences, along with associated 95 % confidence intervals, were calculated along with comparison and global P values where appropriate. Predictors with global P value <0.2 were included in an initial multivariable binary logistic regression model, and backwards elimination was performed until all covariates had a P value <0.2 to generate a final multivariable model [21]. Given there were three outcomes of interest a Bonferroni correction was conducted and alpha set at 0.017. Analyses were repeated for the total cohort, and subgroups of type A aortic dissections and type B aortic dissections respectively. This study is reported in accordance with the Strengthening the Reporting of Observational Studies in Epidemiology (STROBE) statement [22].

## Results

### Study cohort

Between 2001 and 2021, 149 patients with CT-confirmed acute aortic dissection were eligible for inclusion in the present study (see Table 1). Of these, 103 (69.1 %) were Stanford type A and 46 (30.9 %) Stanford type B and their median ADDRS was 2 (IQR: 1–2). In the study cohort, 61.9 % of patients were male with a median age of 67 years (IQR: 53–77), 38.2 % had a smoking history, 71.0 % had a history of hypertension and 65.2 % were ASA IV and above. Majority of patients (70.2 %) arrived via ambulance, 28.2 % self-presented and 1.5 % were airlifted. 51.8 % were treated with surgery, 2.8 % with endovascular intervention, and 45.5 % were managed non-operatively with best medical treatment in a critical care setting. Median operative duration was 420 min (IQR: 330–499), and postoperative complications were 7.7 % Clavien-Dindo Grade I, 35.9 % Grade II, 11.5 % Grade IIIa, 14.1 % Grade IIIb, 7.7 % Grade IVa, 5.1 % Grade IVb, and 18.0 % Grade V. Median hospital length of stay was 10 days (IQR: 5–17). Median first systolic blood pressure recorded at hospital was 128 mmHg (IQR: 104–161). Temporal variables are outlined in Table 2. Numbers and missing data for each variable can be found in the supplement.

**Table 1:** Patient characteristics.

Characteristic	Individuals with Type A Aortic Dissection (*n* = 103)	Individuals with Type B Aortic Dissection (*n* = 46)
Age years - mean (SD)	63.9 (16.5)	65.6 (17.1)
Sex female - number ( %)	43 (41.7)	13 (28.3)
Method hospital arrival ambulance - number ( %)	67 (65.0)	25 (54.3)
Aortic dissection episode 1st dissection - number ( %)	95 (92.2)	37 (80.4)
Background hypertension present - number ( %)	71 (68.9)	32 (69.6)
First systolic BP hospital - mean (SD)	121.9 (35.3)	152.9 (40.1)
Smoking history present - number ( %)	38 (36.9)	14 (30.4)
ASA equal to or greater than IV - number ( %)	75 (72.8)	15 (32.6)
Management approach surgical - number ( %)	71 (68.9)	3 (6.5)
Operative duration minutes - mean (SD)	435.0 (116.9)	270.0 (150.3)
Clavien-Dindo III or greater - number ( %)	41 (39.8)	3 (6.5)
Duration hospital admission - mean (SD)	13.4 (11.3)	10.5 (8.4)
In-hospital mortality dead - number ( %)	30 (29.1)	5 (10.9)
Thirty-day mortality dead - number ( %)	30 (29.1)	5 (10.9)
Six-month mortality dead - number ( %)	31 (30.1)	5 (10.9)

**Table 2:** Descriptive statistics of continuous variables in analysis.

Variable	Median (IQR)
Time from first hospital contact to diagnosis on CT (min)	142 (65–284)
Time from symptom onset to ambulance notification (min)	15 (9–61)
Time from symptom onset to ambulance arrival at scene (min)	22 (17–64)
Time from ambulance dispatch to first hospital contact (min)	56 (47–75)
Time from ambulance notification to triage (min)	61 (48–75)
Time from triage to diagnosis on CT (min)	136.5 (64.5–281.5)
Time from first hospital contact to surgical registrar review (min)	180 (101.5–343)
Time from first hospital contact to consultant surgeon review (min)	191 (95–353)
Time from diagnosis on CT to start of surgery (min)	187.5 (110–364.5)

### In-hospital mortality

Results of the univariate regressions of in-hospital mortality can be found in Table 3. The in-hospital mortality rate was 29.1 % (*n* = 30) for type A dissection vs 10.9 % (*n* = 5) for type B (as summarised in Table 1). Patients with type A dissections had odds of in-hospital mortality 3.3 times greater than those with type B, but did not reach statistical significance (*p* = 0.021). For type A patients, every one-year increase in age increased odds of in-hospital mortality by 4 % (*p* = 0.0076), and odds of in-hospital mortality were 10.9 times greater with conservative management than surgical (*p* < 0.0001). For type B patients, every 10 mmHg decrease in first systolic blood pressure recorded at hospital in comparison to the median increased odds of in-hospital mortality by 29 % (*p* = 0.0396).

**Table 3:** Univariate regressions of in-hospital mortality.

Predictor	Comparison	Odds Ratio (95 % CI)	Comparison P value	Global P value
**Time from first hospital contact to diagnosis on CT**	Per 1 h increase			
Total cohort		0.90 (0.78, 1.05)		0.1769
Type A		0.94 (0.84, 1.05)		0.2752
Type B		0.3 (0.07, 1.34)		0.1142
**Age**	Per 1 year increase			
Total cohort		1.04 (1.01, 1.07)		**0.0074**
Type A		1.04 (1.01, 1.08)		**0.0076**
Type B		1.04 (0.96, 1.11)		0.3308
**Operative duration**	Per 1 h increase			
Total cohort		1.12 (0.85, 1.49)		0.4239
Type A		1.24 (0.91, 1.7)		0.1719
Type B		Did not converge		
**Aortic Dissection Detection Risk Score**	Per 1 unit increase			
Total cohort		1.17 (0.72, 1.9)		0.5271
Type A		0.9 (0.52, 1.54)		0.6971
Type B		3.38 (0.95, 11.95)		0.0592
**First systolic blood pressure**	Per 10 mmHg increase			
Total cohort		0.88 (0.79, 0.99)		**0.0273**
Type A		0.96 (0.84, 1.09)		0.5305
Type B		0.71 (0.51, 0.98)		**0.0396**
**ASA class**	Per 1 unit increase			
Total cohort		2.04 (1.28, 3.25)		**0.0028**
Type A		1.36 (0.86, 2.14)		0.192
Type B		Did not converge		
**Time from symptom onset to ambulance notification**	Per 1 h increase			
Total cohort		0.66 (0.23, 1.88)		0.4325
Type A		0.52 (0.1, 2.85)		0.4518
Type B		0.80 (0.29, 2.26)		0.6763
**Time from symptom onset to ambulance arrival**	Per 1 h increase			
Total cohort		0.57 (0.15, 2.15)		0.4045
Type A		0.33 (0.04, 2.68)		0.3011
Type B		2.18 (0.13, 37.3)		0.5902
**Time from ambulance dispatch to first hospital contact**	Per 1 h increase			
Total cohort		0.95 (0.73, 1.23)		0.703
Type A		0.94 (0.73, 1.22)		0.666
Type B		0.63 (0.05, 7.59)		0.715
**Time from ambulance notification to triage**	Per 1 h increase			
Total cohort		0.97 (0.78, 1.20)		0.7567
Type A		0.97 (0.79, 1.2)		0.8005
Type B		0.61 (0.05, 8.24)		0.7108
**Time from triage to diagnosis on CT**	Per 1 h increase			
Total cohort		0.91 (0.79, 1.05)		0.1958
Type A		0.94 (0.84, 1.05)		0.2799
Type B		0.13 (0.01, 2.22)		0.1568
**Time from first hospital contact to registrar review**	Per 1 h increase			
Total cohort		0.86 (0.69, 1.09)		0.211
Type A		0.96 (0.83, 1.11)		0.5421
Type B		0.61 (0.31, 1.21)		0.1587
**Time from first hospital contact to consultant review**	Per 1 h increase			
Total cohort		0.83 (0.65, 1.07)		0.1544
Type A		0.98 (0.69, 1.39)		0.9115
Type B		0.69 (0.37, 1.27)		0.2307
**Time from diagnosis on CT to start of surgery**	Per 1 h increase			
Total cohort		1.00 (0.97, 1.02)		0.8514
Type A		1.03 (0.98, 1.07)		0.2251
Type B		Did not converge		
**Method of hospital arrival**	Ambulance/airlift vs self-presentation			
Total cohort		2..39 (0.84, 6.81)		0.1037
Type A		1.86 (0.61, 5.63)		0.2738
Type B		Did not converge		
**Stanford class**	Type A vs Type B			
Total cohort		3.33 (1.20, 9.27)		**0.021**
**Management Approach**				
Total cohort	Conservative vs endovascular	1.53 (0.15, 15.63)	0.71775	0.0664
	Conservative vs surgical	2.60 (1.16, 5.81)	**0.0199**	
	Endovascular vs surgical	1.69 (0.16, 17.70)	0.6596	
Type A	Conservative vs surgical	10.87 (3.85, 30.74)		**<0.0001**
Type B	Conservative vs surgical	Did not converge		
**Sex**	Female vs male			
Total cohort		0.88 (0.39, 1.95)		0.7441
Type A		0.53 (0.21, 1.33)		0.1753
Type B		4.50 (0.66, 30.91)		0.1261
**Smoking history**	No vs yes			
Total cohort		1.14 (0.49, 2.62)		0.7642
Type A		0.98 (0.4, 2.42)		0.9651
Type B		Did not converge		
**Hypertension**	No vs yes			
Total cohort		1.06 (0.46, 2.48)		0.8895
Type A		0.95 (0.36, 2.5)		0.9211
Type B		1.76 (0.26, 11.98)		0.5647

Results of the multivariable regressions of in-hospital mortality can be found in the supplement. For the total cohort, patients with type A dissection had odds of in-hospital mortality 3.0 times greater than type B (*p* = 0.0005) and conservative management had odds of in-hospital mortality 2.9 times greater than surgical (*p* < 0.0001).

### 30-day mortality

Results of the univariate regressions of 30-day mortality can be found in Table 4. The 30-day mortality rate was 29.1 % (*n* = 30) for type A dissection vs 10.9 % (*n* = 5) for type B (as summarised in Table 1). For type A patients, every one year increase in age was associated with a 4 % increase in odds of 30-day mortality (*p* = 0.0079), and a nonoperative management approach was associated with 10.3 times greater odds of 30-day mortality than a surgical approach (*p* < 0.0001). For type B patients, every one unit increase in ADDRS was associated with 3.7 times greater odds of 30-day mortality (*p* = 0.0475), and every 10 mmHg decrease in first systolic blood pressure recorded at hospital was associated with a 27 % increase in odds of 30-day mortality (*p* = 0.0496).

**Table 4:** Univariate regressions of 30-day mortality.

Predictor	Comparison	Odds Ratio (95 % CI)	Comparison P value	Global P value
**Time from first hospital contact to diagnosis on CT** Per 1 h increase				
Total cohort		0.90 (0.78, 1.04)		0.1691
Type A		0.94 (0.83, 1.05)		0.2597
Type B		0.31 (0.07, 1.35)		0.1192
**Age**	Per 1 year increase			
Total cohort		1.04 (1.01, 1.07)		**0.0051**
Type A		1.04 (1.01, 1.08)		**0.0079**
Type B		1.05 (0.97, 1.14)		0.2702
**Operative duration**	Per 1 h increase			
Total cohort		1.13 (0.85, 1.5)		0.4027
Type A		1.25 (0.91, 1.7)		0.166
Type B		Did not converge		
**Aortic Dissection Detection Risk Score**	Per 1 unit increase			
Total cohort		1.19 (0.73, 1.94)		0.483
Type A		0.9 (0.53, 1.54)		0.7017
Type B		3.73 (1.01, 13.71)		0.0475
**First systolic blood pressure**	Per 10 mmHg increase			
Total cohort		0.89 (0.79, 0.99)		0.0331
Type A		0.95 (0.84, 1.09)		0.4785
Type B		0.73 (0.53, 1.00)		0.0496
**ASA class**	Per 1 unit increase			
Total cohort		2.06 (1.29, 3.31)		**0.0026**
Type A		1.41 (0.89, 2.24)		0.1462
Type B		Did not converge		
**Time from symptom onset to ambulance notification**	Per 1 h increase			
Total cohort		0.64 (0.22, 1.85)		0.4068
Type A		0.52 (0.1, 2.75)		0.4403
Type B		0.77 (0.27, 2.22)		0.633
**Time from symptom onset to ambulance arrival**	Per 1 h increase			
Total cohort		0.55 (0.15, 2.06)		0.3741
Type A		0.33 (0.04, 2.56)		0.291
Type B		1.96 (0.12, 31.74)		0.6358
**Time from ambulance dispatch to first hospital contact**	Per 1 h increase			
Total cohort		0.95 (0.73, 1.23)		0.6851
Type A		0.94 (0.73, 1.22)		0.6513
Type B		0.69 (0.08, 6.03)		0.7362
**Time from ambulance notification to triage**	Per 1 h increase			
Total cohort		0.96 (0.78, 1.19)		0.7369
Type A		0.97 (0.79, 1.2)		0.777
Type B		0.67 (0.09, 5.15)		0.703
**Time from triage to diagnosis on CT**	Per 1 h increase			
Total cohort		0.91 (0.79, 1.04)		0.1814
Type A		0.94 (0.84, 1.05)		0.2631
Type B		0.15 (0.01, 2.3)		0.1719
**Time from first hospital contact to registrar review**	Per 1 h increase			
Total cohort		0.86 (0.69, 1.08)		0.2008
Type A		0.95 (0.81, 1.11)		0.5197
Type B		0.63 (0.32, 1.24)		0.1826
**Time from first hospital contact to consultant review**	Per 1 h increase			
Total cohort		0.84 (0.65, 1.07)		0.1559
Type A		0.98 (0.69, 1.39)		0.9115
Type B		0.68 (0.36, 1.27)		0.2274
**Time from diagnosis on CT to start of surgery**	Per 1 h increase			
Total cohort		1.00 (0.97, 1.02)		0.8029
Type A		1.02 (0.98, 1.07)		0.2552
Type B		Did not converge		
**Method of hospital arrival**	Ambulance/airlift vs self-presentation			
Total cohort		2.42 (0.84, 6.94)		0.1006
Type A		1.99 (0.65, 6.05)		0.225
Type B		Did not converge		
**Stanford class**	Type A vs Type B			
Total cohort		3.04 (1.09, 8.53)		0.0343
**Management Approach**				
Total cohort	Conservative vs endovascular	1.74 (0.17, 17.73)	0.6414	0.0461
	Conservative vs surgical	2.8 (1.24, 6.31)	**0.0132**	
	Endovascular vs surgical	1.61 (0.15, 16.84)	0.6904	
Type A	Conservative vs surgical	10.31 (3.64, 29.2)		**<0.0001**
Type B	Conservative vs surgical	Did not converge		
**Sex**	Female vs male			
Total cohort		0.91 (0.41, 2.04)		0.8171
Type A		0.55 (0.22, 1.38)		0.2043
Type B		5.06 (0.72, 35.78)		0.104
**Smoking history**	No vs yes			
Total cohort		1.17 (0.5, 2.72)		0.7129
Type A		0.99 (0.4, 2.46)		0.9839
Type B		Did not converge		
**Hypertension**	No vs yes			
Total cohort		1.05 (0.45, 2.46)		0.9128
Type A		0.9 (0.34, 2.36)		0.8227
Type B		1.93 (0.28, 13.44)		0.5086

Results of the multivariable regressions of 30-day mortality can be found in the supplement. Type A patients had 2.8 times greater odds of 30-day mortality compared with type B patients (*p* = 0.0009). For the total cohort, every 10 mmHg decrease in first systolic blood pressure recorded at hospital was associated with a 15 % increase in odds of 30-day mortality (*p* = 0.0489), and conservative management was associated with 2.8 times greater odds of 30-day mortality versus surgical (*p* < 0.0001).

### 6-month mortality

Results of the univariate regressions of 6-month mortality can be found in Table 5. The 6-month mortality rate was 30.1 % (*n* = 31) for type A dissection vs 10.9 % (*n* = 5) for type B (as summarised in Table 1). For type A patients, every one-year increase in age was associated with a 4 % increase in odds of 6-month mortality (*p* = 0.0129), and conservative management was associated with 8.7 times greater 6-month mortality than surgical (*p* < 0.0001). For type B patients, every one unit increase in ADDRS was associated with a 3.7-fold increase in odds of 6-month mortality (*p* = 0.0475), and every 10 mmHg decrease in first systolic blood pressure recorded in hospital was associated with a 27 % increase in odds of 6-month mortality (*p* = 0.0496).

**Table 5:** Univariate regressions of 6-month mortality.

Predictor	Comparison	Odds Ratio (95 % CI)	Comparison P value	Global P value
**Time from first hospital contact to diagnosis on CT**		Per 1 h increase		
Total cohort		0.91 (0.8, 1.04)		0.176
Type A		0.94 (0.85, 1.04)		0.2533
Type B		0.31 (0.07, 1.35)		0.1192
**Age**	Per 1 year increase			
Total cohort		1.04 (1.01, 1.07)		**0.0085**
Type A		1.04 (1.01, 1.07)		**0.0129**
Type B		1.05 (0.97, 1.14)		0.2702
**Operative duration**	Per 1 h increase			
Total cohort		1.1 (0.84, 1.45)		0.4974
Type A		1.19 (0.88, 1.62)		0.2523
Type B		Did not converge		
**Aortic Dissection Detection Risk Score**	Per 1 unit increase			
Total cohort		1.23 (0.76, 2.00)		0.4038
Type A		0.93 (0.54, 1.60)		0.8029
Type B		3.73 (1.01, 13.71)		0.0475
**First systolic blood pressure**	Per 10 mmHg increase			
Total cohort		0.89 (0.79, 0.99)		0.0328
Type A		0.96 (0.84, 1.09)		0.5364
Type B		0.73 (0.53, 1.00)		0.0496
**ASA class**	Per 1 unit increase			
Total cohort		2.13 (1.33, 3.4)		**0.0016**
Type A		1.44 (0.91, 2.28)		0.1177
Type B		Did not converge		
**Time from symptom onset to ambulance notification**	Per 1 h increase			
Total cohort		0.97 (0.86, 1.1)		0.6583
Type A		0.98 (0.87, 1.09)		0.7009
Type B		0.77 (0.27, 2.22)		0.633
**Time from symptom onset to ambulance arrival**	Per 1 h increase			
Total cohort		0.97 (0.87, 1.09)		0.6462
Type A		0.97 (0.86, 1.10)		0.6145
Type B		1.96 (0.12, 31.74)		0.6358
**Time from ambulance dispatch to first hospital contact**	Per 1 h increase			
Total cohort		0.94 (0.73, 1.22)		0.6596
Type A		0.94 (0.72, 1.21)		0.6207
Type B		0.69 (0.08, 6.03)		0.7362
**Time from ambulance notification to triage**	Per 1 h increase			
Total cohort		0.96 (0.77, 1.19)		0.6968
Type A		0.96 (0.78, 1.19)		0.7286
Type B		0.67 (0.09, 5.15)		0.703
**Time from triage to diagnosis on CT**	Per 1 h increase			
Total cohort		0.92 (0.81, 1.04)		0.183
Type A		0.94 (0.85, 1.04)		0.2517
Type B		0.15 (0.01, 2.3)		0.1719
**Time from first hospital contact to registrar review**	Per 1 h increase			
Total cohort		0.90 (0.74, 1.10)		0.315
Type A		0.97 (0.86, 1.08)		0.5596
Type B		0.63 (0.32, 1.24)		0.1826
**Time from first hospital contact to consultant review**	Per 1 h increase			
Total cohort		0.84 (0.65, 1.07)		0.1559
Type A		0.98 (0.69, 1.39)		0.9115
Type B		0.68 (0.36, 1.27)		0.2274
**Time from diagnosis on CT to start of surgery**	Per 1 h increase			
Total cohort		0.99 (0.97, 1.02)		0.6786
Type A		1.02 (0.98, 1.06)		0.3362
Type B		Did not converge		
**Method of hospital arrival**	Ambulance/airlift vs self-presentation			
Total cohort		2.60 (0.90, 7.47)		0.0761
Type A		2.18 (0.71, 6.66)		0.1721
Type B		Did not converge		
**Stanford class**	Type A vs Type B			
Total cohort		3.39 (1.21, 9.50)		0.0202
**Management Approach**				
Total cohort	Conservative vs endovascular	1.74 (0.17, 17.73)	0.6414	0.1094
	Conservative vs surgical	2.36 (1.06, 5.27)	0.0359	
	Endovascular vs surgical	1.36 (0.13, 14.15)	0.7975	
Type A	Conservative vs surgical	8.65 (3.08, 24.30)		<0.0001
Type B	Conservative vs surgical	Did not converge		
**Sex**	Female vs male			
Total cohort		0.87 (0.39, 1.96)		0.7445
Type A		0.52 (0.21, 1.30)		0.1616
Type B		5.06 (0.72, 35.78)		0.104
**Smoking history**	No vs yes			
Total cohort		1.07 (0.47, 2.46)		0.8705
Type A		0.91 (0.37, 2.24)		0.8291
Type B		Did not converge		
**Hypertension**	No vs yes			
Total cohort		0.94 (0.4, 2.2)		0.8829
Type A		0.76 (0.29, 2.01)		0.5853
Type B		1.93 (0.28, 13.44)		5.086

Results of the multivariable regressions of 6-month mortality can be found in the supplement. Type A patients were associated with 2.8 times greater odds of 6-month mortality than type B patients (*p* = 0.0008). For the total cohort, conservative management was associated with 2.5 times greater odds of 6-month mortality than surgical (*p* = 0.0004). For type A patients, conservative management was associated with 9.8 times greater odds of 6-month mortality than surgical (*p* = 0.0014). For type A patients, female sex was associated with 81 % lower odds of 6-month mortality than male sex (*p* = 0.0171).

### Time to diagnosis and treatment

In univariate logistic models for the full cohort, delays in diagnosis and operative intervention showed trends toward lower mortality but were not statistically significant. Specifically, each 1-hour delay to CT diagnosis corresponded to a non-significant 8 % lower odds of in-hospital death (OR 0.92; 95 % CI 0.82–1.03; *p* = 0.143), and each 1-hour delay to surgery to a non-significant 0.2 % lower odds (OR 0.998; 95 % CI 0.98–1.02; *p* = 0.885).

### Survival analysis

Supplementary figure 2 shows Kaplan–Meier curves for in‐hospital survival stratified by management approach. The curves separate early, with surgical patients exhibiting the best survival, conservative patients the worst, and endovascular patients intermediate. A log-rank test confirmed these differences were statistically significant (*p* = 0.018).

### Subgroup logistic regression

Separate multivariable logistic regressions were conducted for each management arm (Supplementary Table 3). Surgical (*n* = 71): none of the predictors - age (OR 1.02, 95 % CI 0.96–1.09; *p* = 0.63), male sex (OR 8.4, 95 % CI 1.2–174; *p* = 0.065), or first systolic BP (OR 1.00, 95 % CI 0.98–1.03; *p* = 0.69) - was significantly associated with in-hospital mortality. Conservative (*n* = 65): each additional year of age increased odds of in-hospital death by 8 % (OR 1.08, 95 % CI 1.03–1.15; *p* = 0.0085), and each 1 mmHg higher first systolic BP was associated with a 3 % reduction in mortality odds (OR 0.97, 95 % CI 0.95–0.99; *p* = 0.0022); sex was not significant (OR 1.77, 95 % CI 0.43–8.40; *p* = 0.447). Endovascular (*n* = 4): sample size too small for reliable multivariable modelling.

## Discussion

This multicentre cohort study characterized the relative impact of clinical and temporal factors on short and mid-term mortality after acute aortic dissection. Within the study population, there was a significant predominance of patients with type A dissections, male sex, non-smoking history, history of hypertension, moderate to severe co-morbidities affecting physical status, and arrival to hospital via ambulance. Increased age and decision to pursue conservative management were significantly associated with increased short and mid-term mortality in type A patients, whereas lower first systolic blood pressure recorded at hospital and higher ADDRS were more significantly associated with increased short and mid-term mortality in type B patients. For type A patients, female sex was associated with substantially lower 6-month mortality.

A notable observation was the increase in-hospital, 30-day and 6-month mortality conveyed by progressively lower first systolic blood pressure recorded at hospital presentation in type B patients. This association is of clinical importance as acute aortic dissection presentations are most commonly associated with a hypertensive state [13]. Given strong association with patient survival in both the short and mid-term, our findings also stress the importance of clinical staff prioritising recording systolic blood pressure early after patient presentation to hospital. Future research should investigate the methods by which blood pressure is recorded in this setting and the pathological process driving the lower blood pressure in acute aortic dissection patients. For type B patients, a hypotensive state may represent progressive extravasation of blood into the false lumen of the aortic dissection, a shocked state from visceral malperfusion, rupture, or cardiogenic shock, and may explain a lower blood pressure and poor survival. In type A patients, a hypotensive state could represent serious cardiac complications such as pericardial tamponade or dissection into the coronary vessels. These blood pressure associations observed in both Type A and B patients acute aortic dissection patients with lower blood pressure may assist decision-making by hospital staff. Hypotension should be identified early, and timely investigations should be undertaken for complications of the dissection that may be causing, or caused by, circulatory shock. Future study should explore whether more focused investigation and resolution of hypotension, or earlier intervention for patients presenting with hypotension is associated with improved outcomes.

Findings from this study benefit clinical decision-making within the early diagnosis and management of acute aortic dissections. Given the large difference in mortality between surgically and conservatively managed type A patients, the clinical threshold for expedited operative intervention should be low for these patients after confirmation of diagnosis on CT. Due to poor outcomes in patients with Type A dissection who are treated conservatively, any patient with a reasonable prospect of survival should be considered for surgery

Multiple associations between patient factors and mortality were described in this study. Increased age was progressively associated with poorer short and mid-term survival. This is likely explained by the consideration that elderly age is usually accompanied by a greater number of comorbidities and accordingly greater mortality [23]. particularly those that are known to be associated with worse outcomes after acute aortic dissection [1]. Evidence for this explanation is provided in the finding that greater ASA class, indicating worse physical status, was also associated with progressively worse survival in the short and mid-term. Within type A patients, female patients experienced substantially lower 6-month mortality when compared with male patients, which adds data to a literature that has contradiction among recent notable publications [[24], [25], [26]]. Further study is required to explore sex differences in aortic dissection outcomes, and also whether international variation exists.

Although neither time‐to‐diagnosis nor time‐to‐operation reached significance—likely due to limited power and the potential for confounding by indication—the consistent trend towards lower mortality with longer intervals may reflect that more stable patients tolerate delays. Nevertheless, rapid diagnostic and treatment pathways remain recommended given the high stakes in acute aortic dissection.

Our Kaplan–Meier analysis confirms that surgical management confers a clear early survival advantage over conservative therapy, with endovascular repair intermediate. The significant log-rank p‐value (0.018) underscores that treatment approach is a major determinant of in‐hospital survival.

The stratified regressions reveal that, in conservatively managed patients, advanced age and hypotension at presentation drive much of the mortality risk (each additional year of age was associated with an 8 % increase in the odds of in-hospital mortality, whereas each 1 mmHg higher initial systolic blood pressure was associated with a 3 % decrease in those odds).In contrast, these factors did not reach significance in the surgical cohort, suggesting that operative intervention may mitigate the prognostic impact of age and initial blood pressure.

These findings reinforce two clinical imperatives: a low threshold for surgical referral (especially in older or hypotensive patients) and aggressive early blood pressure control. Our inability to model the endovascular subgroup reflects limited numbers but indicates the need for larger studies to clarify risk factors in that emerging treatment arm.

The in-hospital mortality rates observed in our cohort (Type A: 29.1 %, Type B: 10.9 %) align closely with figures reported by the International Registry of Acute Aortic Dissection (IRAD). Early IRAD data noted surgical mortality of 26 % for Type A and medical management mortality of 10.7 % for Type B dissections.^4 27^ More recent IRAD analyses, encompassing data up to 2013 and 2018, have shown a significant decrease in-hospital mortality for Type A dissections (from 31 % to 22 % overall, and surgical mortality from 25 % to 18 %), while Type B in-hospital mortality has remained relatively stable (ranging from 12 % to 14 %) [15]. This consistency between our cohort's mortality burden and global trends, particularly for medically managed Type B dissections, suggests a shared clinical challenge.

Consistent with findings from IRAD, our study identified increased age as a significant predictor of mortality, particularly for Type A patients. IRAD data similarly show higher mortality among older patients, with those over 70 years having increased odds of death for Type A dissection [14]. Furthermore, the significantly higher mortality associated with conservative management for Type A dissections in our cohort (OR 10.9 for in-hospital mortality) strongly reinforces IRAD's consistent message that surgical intervention is associated with superior survival for Type A dissections, even in high-risk patients [14]. The observation that lower first systolic blood pressure predicted increased mortality in Type B patients also aligns with IRAD's identification of hypotension/shock as an ominous sign and a strong predictor of mortality, particularly in Type B dissections [28].

Our finding that female sex was associated with substantially *lower* 6-month mortality in Type A patients presents a nuanced contrast to some IRAD analyses. These analyses have reported higher in-hospital mortality for women with Type A dissections (e.g., 33.5 % vs. 24.1 % in men, or 32 % vs. 22 % in men after adjustment for age and hypertension) [14,28]. This discrepancy warrants further discussion regarding potential regional variations, differences in cohort characteristics, or evolving management outcomes over time, and highlights an important area for future research, as our study itself suggests.

While our cohort of 149 patients is smaller than the thousands enrolled in IRAD (e.g., over 11,000 cases from >61 sites across 15 countries), the consistency of our key findings with this large global registry enhances the generalizability and external validity of the present study's conclusions [15].

This study had multiple limitations. It is a retrospective, non-randomised observational study and so it is not possible to draw associations between interventions and outcomes since the decision to operate is often based on assessment of operative risk. However, we included all consecutive patients across multiple metropolitan centres, where data were obtainable and inclusion criteria were met, thereby limiting bias. Limited sample size may have prevented optimal characterization of the association between temporal variables and patient outcomes to add to the existing literature [29]. All patients included within this multicentre cohort study presented to metropolitan centres, thereby limiting potential translatability of study findings to rural and remote settings. When considering study findings regarding management approaches, potential bias may have been added by the fact that conservative management may be pursued as part of palliative care in rapidly deteriorating patients where surgical intervention and the associated physiological stress is likely to be of little benefit to patient outcome. Because palliative patients have an intrinsically higher mortality, our estimated mortality for the conservative strategy may be inflated. Future studies should distinguish between surgical refusal and palliative intent when assessing conservative management outcomes. A paucity of patients were treated using endovascular intervention relative to other prominent reports in the literature [27]. potentially limiting wider translatability. Operation type, operative time, and use of alternative cerebral protection strategies were not included within statistical analysis. Blood pressure was recorded via measurement at the non-invasive brachial artery, which may be misleading in cases of aortic dissection, and accordingly may add bias to the study findings. Further, investigation was not conducted regarding the specific cause of cases of hypotension in this patient cohort, which may be an important avenue of future research. Although the total duration of the data collected in this study spans 20 years, study findings may not be consistent across decades given significant advancements in clinical care and patient outcomes that have occurred [13]. Future research into avoidable factors associated with mortality and adverse clinical outcomes for AAD patients is warranted [[30], [31], [32]]. Finally, although our follow-up extended to 6-months – an interval we classify as mid-term – it does not capture long-term outcomes beyond one year, which will require further study.

## Conclusion

This multicentre cohort study characterized the relative impact of clinical and temporal factors on short and mid-term mortality after acute aortic dissection. Age and decision to pursue conservative management were associated with a significantly higher mortality in type A patients, whereas lower first systolic blood pressure recorded in hospital and a higher Aortic Dissection Detection Risk Score were associated with increased mortality in type B patients. Future research via strong study design is required to further characterize the associations described in this study, particularly those between temporal factors within the diagnosis and management of acute aortic dissection and subsequent patient outcomes.

## CRediT authorship contribution statement

**Joshua G. Kovoor:** Writing – review & editing, Writing – original draft, Project administration, Methodology, Investigation, Formal analysis, Data curation, Conceptualization. **John M. Glynatsis:** Writing – review & editing, Investigation, Data curation. **Nikolaos C. Glynatsis:** Writing – review & editing, Investigation, Data curation. **Domenico Perrotta:** Writing – review & editing, Investigation, Data curation. **Elyssa Chan:** Writing – review & editing, Investigation, Data curation. **Timothy Daniell:** Writing – review & editing, Investigation, Data curation. **Stephen Bacchi:** Writing – review & editing, Methodology, Investigation, Formal analysis, Data curation, Conceptualization. **Brandon Stretton:** Writing – review & editing, Methodology, Investigation, Data curation, Conceptualization. **Daksh Tyagi:** Writing – review & editing, Formal analysis, Data curation. **Joseph N. Hewitt:** Writing – review & editing, Investigation, Data curation. **Angelyn L.W. Khong:** Writing – review & editing, Investigation, Data curation. **Diana U. Siriwardena:** Writing – review & editing, Investigation, Data curation. **David X.H. Ling:** Writing – review & editing, Investigation, Data curation. **Christopher D. Ovenden:** Writing – review & editing, Investigation, Data curation. **Rohan Arasu:** Writing – review & editing, Investigation, Data curation. **Jonathan Henry W. Jacobsen:** Writing – review & editing, Methodology, Investigation, Formal analysis, Data curation. **Suzanne Edwards:** Writing – review & editing, Methodology, Investigation, Formal analysis, Data curation. **Matthew Marshall-Webb:** Writing – review & editing, Supervision, Investigation. **Pramesh Kovoor:** Writing – review & editing, Supervision, Investigation. **Benjamin A.J. Reddi:** Writing – review & editing, Supervision, Investigation. **Justin C.Y. Chan:** Writing – review & editing, Supervision, Investigation. **Fabio Ramponi:** Writing – review & editing, Supervision, Investigation. **Kurian J. Mylankal:** Writing – review & editing, Supervision, Investigation. **Michael G. Worthington:** Writing – review & editing, Supervision, Investigation. **Aashray K. Gupta:** Writing – review & editing, Writing – original draft, Project administration, Methodology, Investigation, Formal analysis, Data curation, Conceptualization.

## Declaration of competing interest

The authors declare that they have no known competing financial interests or personal relationships that could have appeared to influence the work reported in this paper.

## Appendix Supplementary materials

Image, application 1
