# Supplementary material for: Factors affecting acute aortic dissection mortality: A multicentre cohort study
Source: Surg Pract Sci. 2025 Sep 21;23:100311. doi: 10.1016/j.sipas.2025.100311 (PMC12519313; doi:10.1016/j.sipas.2025.100311)
Supplement: Supplementary file 1 [file mmc1.docx]

**SUPPLEMENTARY APPENDIX**

***Supplementary Figure 1. Frequency tables of all categorical variables in analysis***

| *Aortic_dissection_class* | *Frequency* | *Percent* | *Cumulative Frequency* | *Cumulative Percent* |
| --- | --- | --- | --- | --- |
| *Type A* | 103 | 69.13 | 103 | 69.13 |
| *Type B* | 46 | 30.87 | 149 | 100.00 |

| *Inhospital_mortality* | *Frequency* | *Percent* | *Cumulative Frequency* | *Cumulative Percent* |
| --- | --- | --- | --- | --- |
| *Alive* | 112 | 76.19 | 112 | 76.19 |
| *Dead* | 35 | 23.81 | 147 | 100.00 |
| *Frequency Missing = 2* | | | | |

| *Thirtyday_mortality* | *Frequency* | *Percent* | *Cumulative Frequency* | *Cumulative Percent* |
| --- | --- | --- | --- | --- |
| *Alive* | 104 | 74.82 | 104 | 74.82 |
| *Dead* | 35 | 25.18 | 139 | 100.00 |
| *Frequency Missing = 10* | | | | |

| *Sixmonth_mortality* | *Frequency* | *Percent* | *Cumulative Frequency* | *Cumulative Percent* |
| --- | --- | --- | --- | --- |
| *Alive* | 99 | 73.33 | 99 | 73.33 |
| *Dead* | 36 | 26.67 | 135 | 100.00 |
| *Frequency Missing = 14* | | | | |

| *Clavien_Dindo_classification_postop_complications* | | | | |
| --- | --- | --- | --- | --- |
| *Clavien_Dindo_classification_pos* | *Frequency* | *Percent* | *Cumulative Frequency* | *Cumulative Percent* |
| *Grade I* | 6 | 7.69 | 6 | 7.69 |
| *Grade II* | 28 | 35.90 | 34 | 43.59 |
| *Grade IIIa* | 9 | 11.54 | 43 | 55.13 |
| *Grade IIIb* | 11 | 14.10 | 54 | 69.23 |
| *Grade IVa* | 6 | 7.69 | 60 | 76.92 |
| *Grade IVb* | 4 | 5.13 | 64 | 82.05 |
| *Grade V* | 14 | 17.95 | 78 | 100.00 |
| *Frequency Missing = 71* | | | | |

| *Clavien_Dindox* | *Frequency* | *Percent* | *Cumulative Frequency* | *Cumulative Percent* |
| --- | --- | --- | --- | --- |
| *1* | 6 | 7.69 | 6 | 7.69 |
| *2* | 28 | 35.90 | 34 | 43.59 |
| *3* | 9 | 11.54 | 43 | 55.13 |
| *4* | 11 | 14.10 | 54 | 69.23 |
| *5* | 6 | 7.69 | 60 | 76.92 |
| *6* | 4 | 5.13 | 64 | 82.05 |
| *7* | 14 | 17.95 | 78 | 100.00 |
| *Frequency Missing = 71* | | | | |

| *Method_Hospital_Arrival* | *Frequency* | *Percent* | *Cumulative Frequency* | *Cumulative Percent* |
| --- | --- | --- | --- | --- |
| *Ambulance* | 92 | 70.23 | 92 | 70.23 |
| *MedSTAR Flight* | 2 | 1.53 | 94 | 71.76 |
| *Self-presentation to ED* | 37 | 28.24 | 131 | 100.00 |
| *Frequency Missing = 18* | | | | |

| *Method_Hospital_Arrivalx* | *Frequency* | *Percent* | *Cumulative Frequency* | *Cumulative Percent* |
| --- | --- | --- | --- | --- |
| *Ambulance/flight* | 94 | 71.76 | 94 | 71.76 |
| *Self presentation to ED* | 37 | 28.24 | 131 | 100.00 |
| *Frequency Missing = 18* | | | | |

| *Management_approach* | *Frequency* | *Percent* | *Cumulative Frequency* | *Cumulative Percent* |
| --- | --- | --- | --- | --- |
| *Conservative* | 65 | 45.45 | 65 | 45.45 |
| *Endovascular* | 4 | 2.80 | 69 | 48.25 |
| *Surgical* | 74 | 51.75 | 143 | 100.00 |
| *Frequency Missing = 6* | | | | |

| *ASA_classification* | *Frequency* | *Percent* | *Cumulative Frequency* | *Cumulative Percent* |
| --- | --- | --- | --- | --- |
| *I* | 3 | 2.17 | 3 | 2.17 |
| *II* | 9 | 6.52 | 12 | 8.70 |
| *III* | 36 | 26.09 | 48 | 34.78 |
| *IV* | 38 | 27.54 | 86 | 62.32 |
| *V* | 51 | 36.96 | 137 | 99.28 |
| *VI* | 1 | 0.72 | 138 | 100.00 |
| *Frequency Missing = 11* | | | | |

| *ASA_classificationx* | *Frequency* | *Percent* | *Cumulative Frequency* | *Cumulative Percent* |
| --- | --- | --- | --- | --- |
| *1* | 3 | 2.17 | 3 | 2.17 |
| *2* | 9 | 6.52 | 12 | 8.70 |
| *3* | 36 | 26.09 | 48 | 34.78 |
| *4* | 38 | 27.54 | 86 | 62.32 |
| *5* | 51 | 36.96 | 137 | 99.28 |
| *6* | 1 | 0.72 | 138 | 100.00 |
| *Frequency Missing = 11* | | | | |

| *Sex* | *Frequency* | *Percent* | *Cumulative Frequency* | *Cumulative Percent* |
| --- | --- | --- | --- | --- |
| *Female* | 56 | 38.10 | 56 | 38.10 |
| *Male* | 91 | 61.90 | 147 | 100.00 |
| *Frequency Missing = 2* | | | | |

| *Smoking_history* | *Frequency* | *Percent* | *Cumulative Frequency* | *Cumulative Percent* |
| --- | --- | --- | --- | --- |
| *No* | 84 | 61.76 | 84 | 61.76 |
| *Yes* | 52 | 38.24 | 136 | 100.00 |
| *Frequency Missing = 13* | | | | |

| *Background_hypertension* | *Frequency* | *Percent* | *Cumulative Frequency* | *Cumulative Percent* |
| --- | --- | --- | --- | --- |
| *No* | 42 | 28.97 | 42 | 28.97 |
| *Yes* | 103 | 71.03 | 145 | 100.00 |
| *Frequency Missing = 4* | | | | |

***Multivariable linear regressions of In hospital mortality versus various predictors***

| *Predictor* | *Comparison* | *Odds Ratio* (95% CI)* | *Global P value* |
| --- | --- | --- | --- |
| Aortic_dissection_classs | Type A vs Type B | 2.95 (1.30, 4.60) | 0.0005 |
| First_systolic_BP_hospital | per 10 unit increase | 0.85 (0.72, 1.00) | 0.0542 |
| Management_approachx | Conservative vs Surgical | 2.89 (1.49, 4.30) | <.0001 |
| time_first_hosp_to_diagnosis_CT | per 1 hour increase | 0.92 (0.82, 1.04) | 0.1782 |

*Modelling the probability that In house mortality=’Death’

***Type A: Multivariable binary logistic regression of In hospital mortality versus various predictors***

| *Predictor and comparison* | *Odds Ratio* (95% CI)* | *P value* |
| --- | --- | --- |
| ASA_classificationx per 1 unit | 0.46 (0.10, 2.08) | 0.3124 |
| Management_Approach Conservative versus Surgical | 22079 (0.03, 1.72E10) | 0.1484 |
| time_first_hosp_to_con_review per 1 hour | 7.26 (0.51, 104.09) | 0.1446 |
| time_first_hosp_to_diagnosis_CT per 1 hour | 0.05 (0.00, 5.59) | 0.2139 |

*Modelling the probability that In hospital mortality=”Yes”

***Type B: Multivariable binary logistic regressions of In hospital mortality versus various predictors***

| *Predictor and comparison* | *Odds Ratio* (95% CI)* | *P value* |
| --- | --- | --- |
| First_systolic_BP_hospital per 10 units | 0.38 (0.07, 1.93) | 0.2425 |
| time_first_hosp_to_diagnosis_CT per 1 hour | 0.37 (0.07, 1.99) | 0.2478 |

*Modelling the probability that In hospital mortality=”Yes”

***Multivariable linear regressions of 30-day mortality versus various predictors***

| *Predictor* | *Comparison* | *OR* (95% CI)* | *Global P value* |
| --- | --- | --- | --- |
| Aortic_dissection_class | Type A vs Type B | 2.82 (1.16, 4.48) | 0.0009 |
| First_systolic_BP_hospital | per 10 unit increase | 0.85 (0.72, 1.00) | 0.0489 |
| Management_approachx | Conservative vs Surgical | 2.80 (1.40, 4.20) | <.0001 |
| time_first_hosp_to_diagnosis_CT | per 1 hour increase | 0.92 (0.82, 1.04) | 0.1755 |

*Modelling the probability that 30-day mortality=’Death’

***Type A: Multivariable binary logistic regression of Thirty day mortality versus various predictors***

| *Outcome and Comparison* | *Odds ratio* (95% CI)* | *P value* |
| --- | --- | --- |
| ASA_classificationx per 1 units | 1.28 (0.64, 2.55) | 0.4925 |
| Age_years per 1 year | 0.98 (0.93, 1.02) | 0.3319 |
| Management_Approach Conservative vs Surgical | 5.45 (0.20, 146.12) | 0.3126 |
| Operative_duration_minutes per 10 units | 1.03 (0.97, 1.09) | 0.3713 |

*Modelling the probability that thirty day mortality=”Yes”

***Type B: Multivariable binary logistic regression of Thirty day mortality versus various predictors***

| *Outcome and Comparison* | *Odds ratio* (95% CI)* | *P value* |
| --- | --- | --- |
| First_systolic_BP_hospital per 10 units | 0.39 (0.08, 1.98) | 0.2572 |
| time_first_hosp_to_diagnosis_CT per 1 hour | 0.38 (0.07, 2.05) | 0.2597 |

*Modelling the probability that thirty day mortality=”Yes”

***Multivariable binary logistic regressions of 6-month mortality versus various predictors***

| *Predictor* | *Comparison* | *Odds ratio* (95% CI)* | *Global P value* |
| --- | --- | --- | --- |
| Aortic_dissection_class | Type A vs Type B | 2.80 (1.16, 4.44) | 0.0008 |
| First_systolic_BP_hospital | per 10 unit increase | 0.86 (0.73, 1.01) | 0.0580 |
| Management_approach | Conservative vs Surgical | 2.48 (1.12, 3.84) | 0.0004 |
| time_first_hosp_to_diagnosis_CT | per 1 hour increase | 0.93 (0.84, 1.03) | 0.1837 |

*Modelling the probability that 30-day mortality=’Death’

***Type A: Multivariable binary logistic regression of Six month mortality versus various predictors***

| *Outcome and Comparison* | *Odds ratio* (95% CI)* | *P value* |
| --- | --- | --- |
| ASA_classificationx per 1 units | 1.50 (0.85, 2.65) | 0.1601 |
| Age_years per 1 year | 1.02 (0.97, 1.06) | 0.4247 |
| Management_Approach Conservative versus Surgical | 9.80 (2.41, 39.94) | 0.0014 |
| Sex Female vs Male | 0.19 (0.05, 0.75) | 0.0171 |

*Modelling the probability that six month mortality=”Yes”

***Type B: Multivariable binary logistic regression of Six month mortality versus various predictors***

| *Outcome and Comparison* | *Odds ratio* (95% CI)* | *P value* |
| --- | --- | --- |
| First_systolic_BP_hospital per 10 units | 0.39 (0.08, 1.98) | 0.2572 |
| time_first_hosp_to_diagnosis_CT per 1 hour | 0.38 (0.07, 2.05) | 0.2597 |

*Modelling the probability that six month mortality=”Yes”

***Univariate logistic regression of time intervals on in-hospital mortality***

| *Predictor* | *OR* | *95% CI* | *p-value* |
| --- | --- | --- | --- |
| Time to CT diagnosis (per hour) | 0.92 | 0.82-1.03 | 0.143 |
| Time from CT to Surgery (per hour) | 0.998 | 0.98-1.02 | 0.885 |

***Supplementary Figure 2. Kaplan-Meier survival curves for the various treatments***


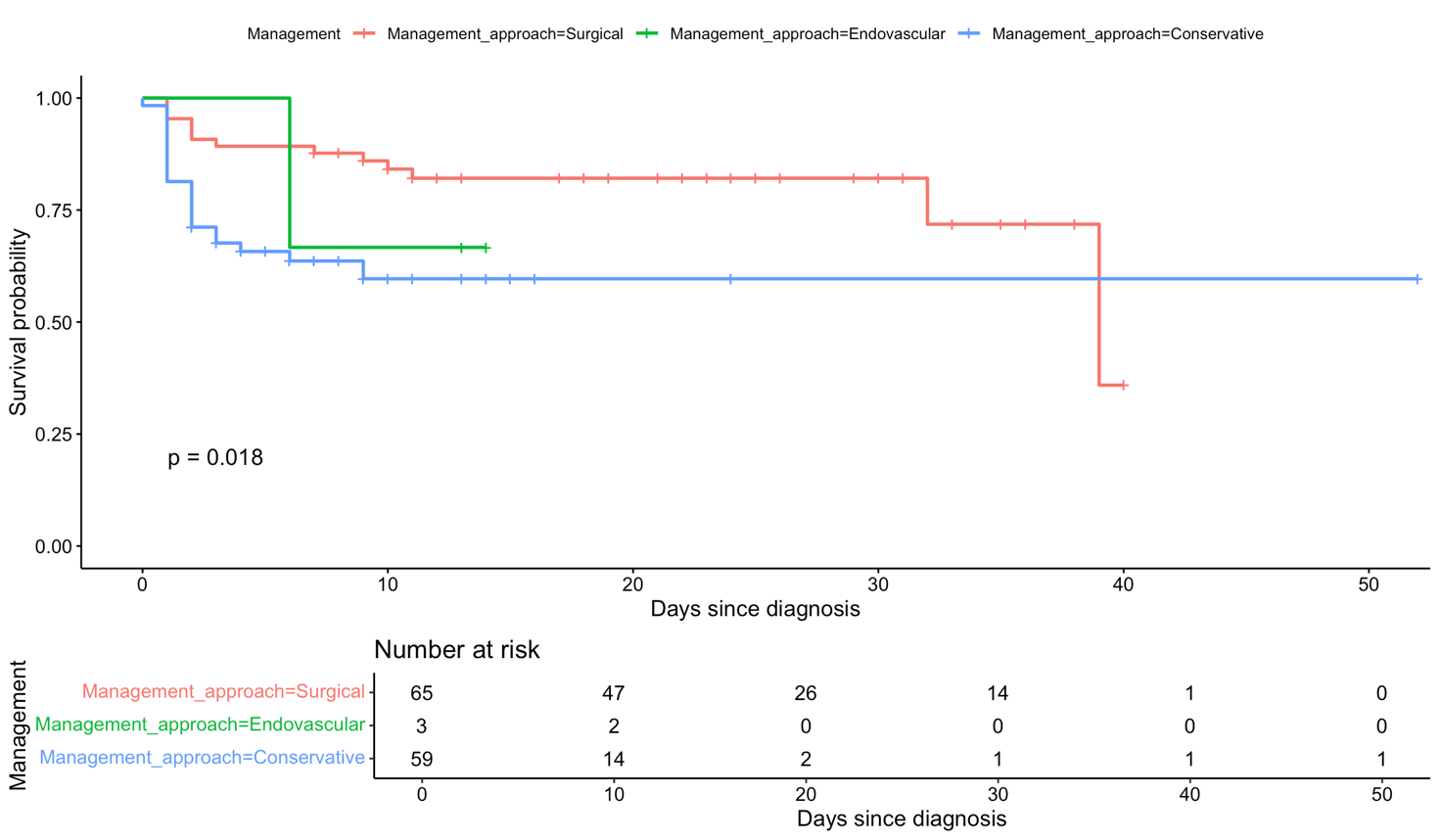


***Supplementary Figure 3. Subgroup Analysis by management approach***

| *Subgroup* | *Predictor* | *Odds Ratio (OR)* | *95% CI* | *p-value* |
| --- | --- | --- | --- | --- |
| Surgical | Age (per year) | 1.02 | 0.96-1.09 | 0.632 |
|  | Male vs Female | 8.42 | 1.24-174.0 | 0.065 |
|  | SBP (mmHg) | 1.00 | 0.98-1.03 | 0.691 |
| Conservative | Age (per year) | 1.08 | 1.03-1.15 | 0.0085 |
|  | Male vs Female | 1.77 | 0.43-8.40 | 0.447 |
|  | SBP (mmHg) | 0.97 | 0.95-0.99 | 0.0022 |
| Endovascular | - | - | - | - |

*Endovascular arm excluded due to insufficient events (n=4)*
